# Interaction of CD99 and its ligand upregulates IL-6 and TNF-α upon T cell activation

**DOI:** 10.1371/journal.pone.0217393

**Published:** 2019-05-23

**Authors:** Nuchjira Takheaw, Papawadee Earwong, Witida Laopajon, Supansa Pata, Watchara Kasinrerk

**Affiliations:** 1 Division of Clinical Immunology, Department of Medical Technology, Faculty of Associated Medical Sciences, Chiang Mai University, Chiang Mai, Thailand; 2 Biomedical Technology Research Center, National Center for Genetic Engineering and Biotechnology, National Science and Technology Development Agency at the Faculty of Associated Medical Sciences, Chiang Mai University, Chiang Mai, Thailand; Universite Paris-Sud, FRANCE

## Abstract

CD99 has been reported to be involved in T cell regulation. CD99 ligand involvement in the regulation of T cell activation has been postulated. In this study, recombinant CD99 proteins were produced and used as a tool for determining the role of CD99 and its ligand interaction. Recombinant CD99 proteins induced the upregulation of IL-6 and TNF-α expression, but not IFN-γ, in anti-CD3 monoclonal antibody activated T cells. The cytokine alteration was not observed in unstimulated T cells indicating the cytokine upregulation required the signal from T cell activation. The upregulation of IL-6 and TNF-α was, in addition, observed in CD3^-^ mononuclear cell population including monocytes and NK cells. The recombinant CD99 proteins, however, did not affect either CD25, CD69 or MHC class II expression or T cell proliferation, upon T cell activation. The CD99 ligands were demonstrated to be expressed on monocytes, NK cells and dendritic cells, but not on B and T cells. Our results indicated the presence of CD99 ligands on leukocyte surface. Interaction between CD99 and its ligands involves the regulation of cytokine production.

## Introduction

Over the last several decades, ligands of several leukocyte surface molecules involving T cell regulation have been identified [1–3]. Uncovering these ligands is essential for understanding the precise immunoregulation mechanism [4]. In the accomplishment of this, the discovery of various leukocyte surface molecules and its ligands interaction will lead to the development of new approaches for treatment of various diseases, including inflammatory diseases and cancers. The PD-1/PD-L1 immune checkpoint blockage in cancer therapy [5–7], the interfering CD28 and CD80/CD86 binding with CTLA-4-Ig in the treatment of rheumatoid arthritis [8, 9] and using anti-CTLA-4 monoclonal antibody (mAb) for cancer treatment [5, 6, 10] are the best examples.

CD99 is a type I integral membrane protein having heavy O-glycosylation [11]. This molecule is broadly expressed on hematopoietic and non-hematopoietic cells [12–17]. CD99 has been demonstrated to play a key role in several biological processes including cell adhesion, differentiation, migration and apoptosis [18–21]. Involvement of CD99 in various cellular processes associated with inflammation, signal transduction and cytokine production was also reported [13, 22–25]. Importantly, CD99 molecule was suggested to function as either the activating or inhibitory receptor in T cell regulation [26–31]. The mechanism of CD99 involving T cell activation, however, remains unclear. For understanding the function of CD99 in T cell regulation, the identification of CD99 ligands expressed on leukocytes is essential [31].

In the present study, we demonstrated that the CD99 ligands were in existence. The CD99 ligands were expressed on monocytes, NK cells and dendritic cells. Interaction between CD99 and its ligands regulated the production of pro-inflammatory cytokines, IL-6 and TNF-α.

## Materials and methods

### Antibodies, reagents and cells

Anti-CD99 mAbs (clone MT99/3, IgG2a) and FITC-conjugated anti-hemoglobin γ-chain mAb (Thal N/B, IgG1) were produced in our laboratory [13, 32]. Anti-CDε mAb (clone OKT3) was obtained from Ortho Pharmaceuticals (Raritan, NJ, USA). FITC-conjugated anti-CD14 (clone M5E2), FITC-conjugated anti-CD19 mAb (clone HIBI9), PerCP-conjugated anti- CD3 mAb (clone UCHT1), PerCP-conjugated anti-CD14 mAb (clone HCD14), Phycoerythrin (PE)-conjugated anti-IL-6 mAb (clone MQ2-13A5), PE-conjugated anti-TNF-α mAb (clone Mab11) and PE-conjugated anti-IFN-γ mAb (clone B27), PE-conjugated anti-IL-4 mAb (clone 8D4-8) and PE-conjugated anti-IL-10 mAb (clone JES3-9D7) were purchased from BioLegend (San Diego, CA, USA). PE/Cy5-conjugated anti-CD3 mAb (clone B159), PE/Cy5-conjugated anti-CD56 mAb (clone HCD56), PE/Cy7-conjugated anti-CD19 mAb (clone HIBI9), PE/Cy7-conjugated anti-CD16 mAb (clone B73.1), FITC-conjugated anti-CD25 mAb (clone M-A251) and FITC-conjugated anti-CD3 mAb (clone UCHT1) were obtained from BD Bioscience (San Jose, CA, USA). PE-conjugated anti-CD69 mAb (clone FN50), FITC-conjugated anti-HLA-DR mAb (clone LT-DR) and PE-conjugated IgG isotype-matched control mAb were purchased from ImmunoTools (Friesoythe, Germany). Horseradish peroxidase (HRP)-conjugated rabbit anti-mouse immunoglobulins (Igs) antibody and HRP-conjugated rabbit anti-human Igs antibody were bought from Dako (Glostrup, Denmark). Streptavidin-conjugate PE was purchased from Molecular Probes (Eugene, OR, USA). Human IgG (HIgG) was prepared by purifying human AB serum using HiTrap Protein G column (GE Healthcare, Uppsala, Sweden).

Lipofectamine 2000 reagent was obtained from Invitrogen (Carlsbad, CA, USA). DTSSP (3,3´-dithiobis[sulfosuccinimidylpropionate]) was acquired from Pierce (Rockford, IL, USA). Brefeldin A, monensin and CFSE (carboxyfluorescein succinimidyl ester) were purchased from Sigma-Aldrich (St. Louis, MO, USA).

HEK293T cells were maintained in DMEM containing 10% fetal bovine serum (FBS) (Gibco, Grand Island, NY, USA), 40 mg/ml gentamicin and 2.5 mg/ml amphotericin B (10%FBS-DMEM) and cultured in a humidified atmosphere of 5% CO_2_ at 37°C. Peripheral blood mononuclear cells (PBMCs) were isolated from healthy donors using Ficoll-Hypaque (IsoPrep) (Robbins Scientific Corporation, Sunnyvale, CA, USA) gradient centrifugation. Briefly, heparinized whole blood was mixed with phosphate buffered saline (PBS) at 1:1 ratio. This diluted blood was overlaid onto Ficoll-Hypaque solution and then spun at 400×g, 25°C for 30 min with break-off setting. After centrifugation, the PBMCs were harvested from white ring at the interphase of Ficoll-Hypaque and plasma layer. The study was approved by the ethics committees of the Faculty of Associated Medical Sciences, Chiang Mai University (AMSEC-58EX-093).

### Construction of plasmid vector harboring gene encoding human CD99HIgG Fc fusion protein

The gene encoding extracellular part of CD99 was amplified from the plasmid DNA pCDM8-CD99 [13] by polymerase chain reaction (PCR). The primers used were designed based on CD99 sequence from NCBI database (gene ID: 4267) and contained NcoI and BgLII cleavage sites: sense 5’-GAGGAGCCATGGATGGTGGTTTCGATTTATCCGAT-3’ and antisense 5’-GAGGAGAGATCTGTCGGCCTCTTCCCCTT-3’. The PCR products were digested with NcoI and BgLII restriction enzymes and ligated into the eukaryotic expression vector pFuse-hIgG1 Fc2 (InvivoGen, Toulouse, France). The inserted CD99 gene was verified by DNA sequencing (First BASE, Selangor, Malaysia).

### Generation of stable CD99HIgG expressing cells

The HEK293T cell line was used as a host cell. The CD99HIgG encoding vectors were transfected into HEK293T cells using Lipofectamine 2000 reagent according to manufacturer protocol. Briefly, 4x10^5^ HEK293T cells in 1 ml of 10% FBS-DMEM were plated into 24-well plate and incubated at 37°C in a 5% CO_2_ incubator overnight. The mixture of plasmid vector and lipofectamine reagent was prepared in DMEM at 1 μg of plasmid to 2 μl of lipofectamine then incubated at room temperature for 20 min. The mixture was gently added into plated HEK293T cells and incubated at 37°C in a 5% CO_2_ for 72 h. For selection and stable expression, transfected HEK293T cells were cultured at 1,000 cells in 150 μl of 10% FBS-DMEM with 100 μg/ml of zeocin drug (InvivoGen). After drug selection, stable CD99HIgG expressing HEK293T cells were cultured in 10% FBS-DMEM containing 50 μg/ml zeocin at 37°C in a 5% CO_2_ incubator. After 80% confluence of cells in 75T flask, culture media containing FBS were washed out and changed to CHO-S-SFM II media (Gibco). Cells were cultured in serum-free media containing 50 μg/ml zeocin at 37°C in 5% CO_2_ for 3 days. Culture supernatant was collected and subjected to purify CD99HIgG (CD99 fused with human IgG Fc part) by affinity chromatography using HiTrap Protein G column (GE Healthcare). The purified recombinant proteins were dialyzed with phosphate buffered saline (PBS). The concentration of CD99HIgG protein was measured by BCA protein assay kit (Pierce). The purified CD99HIgG protein was analyzed by ELISA and Western blotting.

### ELISA for detection of CD99HIgG

Purified CD99HIgG or CD147Rg (control) [33] (1 μg/ml; 50 μl) were coated into 96-well ELISA plates (Costar, NY, USA). The plates were washed and surface blocked with 2% skimmed milk in PBS. To detect CD99 protein, anti-CD99 mAb (MT99/3) was added, followed by HRP-conjugated rabbit anti-mouse Igs antibody. To detect human IgG part, HRP-conjugated rabbit anti-human Igs antibody was added. After incubation, 3,3’,5,5’-Tetramethylbenzidine (TMB) substrate was added. The reaction was stopped using 1M HCl and the absorbance was measured at 450 nm.

### Western blotting for characterization of CD99HIgG

5 μg of purified CD99HIgG in non-reducing and reducing conditions with 1% dithiothreitol (DTT) were resolved by 10% SDS-PAGE and subsequently transferred to a PVDF membrane (Merck Millipore, Darmstadt, Germany). The membranes were blocked with 5% skimmed milk and incubated with either anti-CD99 mAb (MT99/3) followed by HRP-conjugated rabbit anti-mouse Igs antibody or HRP-conjugated rabbit anti-human Igs antibody. After incubation step, the membranes were washed and then the reaction bands were developed using chemiluminescent Western blotting detection reagent (ECL Western Blotting Substrate) (Pierce).

### Study of CD99 and CD99 ligand interaction on cytokine production

PBMCs were cultured with or without immobilized anti-CD3 mAb (OKT3) at 25 ng/ml in the presence or absence of purified CD99HIgG or HIgG control (5 μg/ml; 250 μl). After cultivation for 1 h at 37°C in a 5% CO_2_ incubator, protein transport inhibitors (1 μg/ml brefeldin A and 1μM monensin) were added and continuously incubated at 37°C in a 5% CO_2_ incubator for 5 h. Cells were stained with cocktail antibody for analysis of monocytes, T cells and B cells or NK cells using FITC-conjugated anti-CD14 mAb, PE/Cy5-conjugated anti-CD3 mAb and PE/Cy7-conjugated anti-CD19 mAb or FITC-conjugated anti-CD3 mAb together with PE/Cy5-conjugated anti-CD56 mAb, respectively. Intracellular staining was performed using 4% paraformaldehyde and 0.1% saponin for cell fixation and permeabilization. The intracellular cytokines were determined using PE-conjugated anti-IL-6, anti-TNF-α, anti-IFN-γ mAb, anti-IL-4 or anti-IL-10 mAbs and detected by BD Accuri C6 flow cytometer (BD Biosciences) or BD FACS Melody cell sorter (BD Biosciences). FACS data were analyzed with FlowJo software.

### Study of CD99 and CD99 ligand interaction on T cell proliferation

PBMCs were labeled with CFSE and CFSE-labeled cells were plated into 96-well-plate with or without immobilized anti-CD3 mAb (OKT3) (25 ng/ml) in the presence or absence of CD99HIgG or HIgG control (5 μg/ml or 10 μg/ml; 100 μl). The cells were incubated at 37°C in a 5% CO_2_ incubator for 3 and 5 days. After cultivation, cells were harvested and cell proliferation was measured by BD Accuri C6 flow cytometer (BD Biosciences) and analyzed with FlowJo software.

### Study of CD99 and CD99 ligand interaction on activation-associated marker expression

PBMCs were incubated with or without immobilized anti-CD3 mAb (OKT3) (25 ng/ml) in combination with or without purified CD99HIgG or HIgG (5 μg/ml; 500 μl). The cells were incubated at 37°C in a 5% CO_2_ incubator. After incubation for 24 and 72 h, cells were collected and stained with PE-anti-CD69 mAb, FITC-anti-CD25 mAb, FITC-anti-HLA-DR mAb, PE-conjugated IgG, or FITC-conjugated Thal N/B (IgG1) isotype-matched control mAbs. The expression of all activation-associated markers was determined by a BD Accuri C6 flow cytometer (BD Biosciences) and analyzed with FlowJo software.

### Immunofluorescence staining with DTSSP crosslinking for determining CD99 ligand

CD99HIgG and irrelevant Fc fusion protein were biotinylated using EZ-Link Sulfo-NHS-LC-Biotin (Thermoscientific, Rockford, lL, USA) according to the manufacturer's protocol. Briefly, 100 μg of recombinant proteins in PBS were mixed with 10-fold molar excess of biotin reagent then incubated at room temperature for 30 min. The dialysis in PBS was performed to remove the excess non-reacted biotin in solution. The irrelevant Fc fusion protein termed CD147ExHIgG was generated in our laboratory, carried out in a similar procedure as CD99HIgG. CD147ExHIgG is an extracellular part of CD147, which was amplified from pCDM8-CD147 [34] by PCR. The primers containing NcoI and BgLII cleavage sites: sense 5’- GAGGAGCCATGGAACCCG GCACAGTCTTCACTA -3’ and antisense 5’- GAGGAGAGATCTCTCGGGGAGGAAGAC GCA -3’ designed based on CD147 sequence from NCBI database (gene ID: 682) were used. The PCR products were then ligated into the pFuse-hIgG1 Fc2 vector at NcoI and BgLII restriction enzymes digestion. This irrelevant Fc fusion protein was also produced in HEK293T cells.

Fc receptor expressed on PBMCs was blocked with 20% human serum (blood group AB) and stained with 20 μg/ml of biotinylated CD99HIgG or biotinylated irrelevant Fc fusion protein for 1 h on ice. After that, 2mM DTSSP were added in order to covalent link bound CD99 recombinant protein and its putative ligands [35, 36]. After incubation on ice for 2 h, 20mM Glycine in PBS were used for neutralizing the excess DTSSP and washed. The bound biotinylated proteins were detected with PE-streptavidin. The surface marker membrane proteins were stained with cocktail antibody for analysis of monocytes and B cells using FITC-anti-CD19 mAb and PerCP-anti-CD14 mAb or T cells and NK cells using FITC-anti-CD3 mAb and PE/Cy5-anti-CD56 mAb or dendritic cell using PerCP-anti-CD14 mAb, PE/Cy5-anti-CD56 mAb, PE/Cy7-conjugated anti-CD3, anti-CD19 and anti-CD16 and FITC-anti-HLA-DR mAb. The stained cells were fixed with 1% paraformaldehyde and analyzed by flow cytometry (BD Accuri C6) or BD FACS Melody cell sorter (BD Biosciences). FACS data were analyzed with FlowJo software.

### Statistical analysis

Statistical analysis was carried out with GraphPad Prism 6 software (San Diego, CA, USA). Significant values were analyzed using unpaired t test and one-way ANOVA with multiple comparison. P-values ≤ 0.05 were considered significant.

## Results

### Production of dimeric form of human soluble CD99-IgG fusion proteins

**Fig 1:**
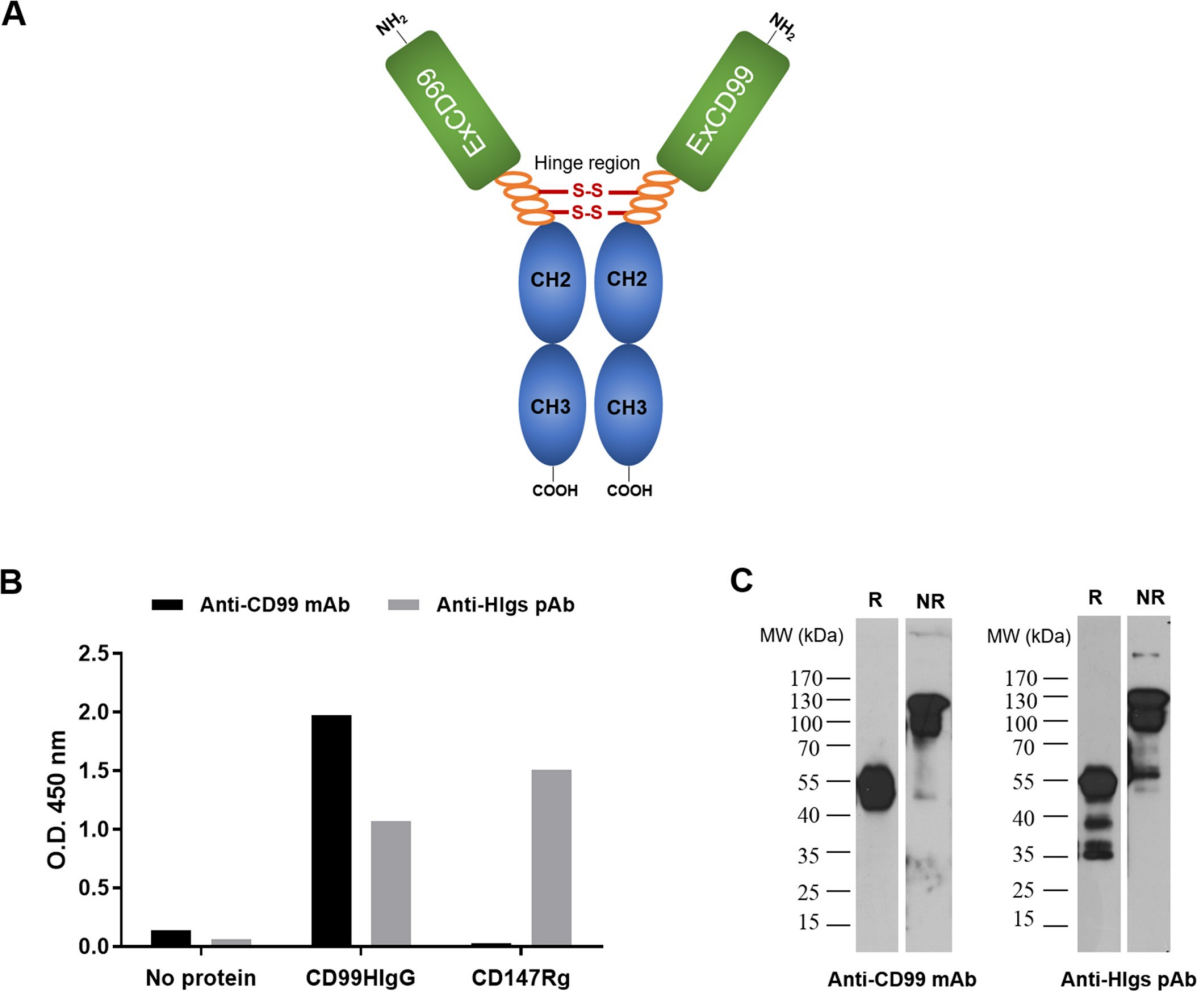
Schematic of constructed recombinant human CD99HIgG fusion protein. (A) Schematic drawing of CD99HIgG construct. Fc portion of human IgG attached to CD99 extracellular part (ExCD99) with hinge region is shown. (B) ELISA was performed for verifying the CD99HIgG construct. The reactivity of anti-CD99 mAb and anti-human Igs (HIgs) pAb to uncoated plate (No protein), purified CD99HIgG and CD147Rg (recombinant protein control) are shown. (C) Western blot analysis of purified CD99HIgG under reducing (R) and non-reducing (NR) conditions using anti-CD99 mAb and anti- human Igs (HIgs) pAb is shown.

To study the role of the interaction of CD99 and its ligand, we firstly generated CD99 recombinant protein fused with Fc part of human IgG in dimeric form (named CD99HIgG) (Fig 1A). The plasmid vector harboring extracellular part of CD99 linked with human IgG Fc part with hinge regions containing disulfide bonds was constructed. By DNA sequencing, the inserted CD99 gene in the vector was 100% identical to the DNA sequence of CD99, gene ID: 4267. Subsequently, the CD99HIgG proteins were expressed in HEK293T cell line as secretory proteins and further purified through protein G column. Purified CD99HIgG was verified by ELISA and western blotting using anti-CD99 mAb (MT99/3) and anti-human Igs pAb. By ELISA, the produced CD99HIgG was specifically recognized by anti-CD99 mAb and anti-human Igs pAb (Fig 1B). Western blot analysis using either anti-CD99 mAb or anti-human Igs pAb showed the same reactivity patterns. In reducing conditions, the CD99HIgG at approximately 50 kDa, which was the monomeric form of CD99HIgG that contained CD99 extracellular part (25 kDa) and Fc portion of human IgG (25 kDa), was observed (Fig 1C). The band at approximately 100 kDa corresponding to dimeric form of CD99HIgG was obtained in non-reducing condition (Fig 1C).

The results indicated that dimeric form of human soluble CD99HIgG fusion proteins were successfully produced by human cell line. The produced CD99HIgG was used as representative of CD99 molecule for studying the interaction to its ligand.

### Interaction of CD99 and its ligand upregulates IL-6 and TNF-α production upon T cell activation

**Fig 2:**
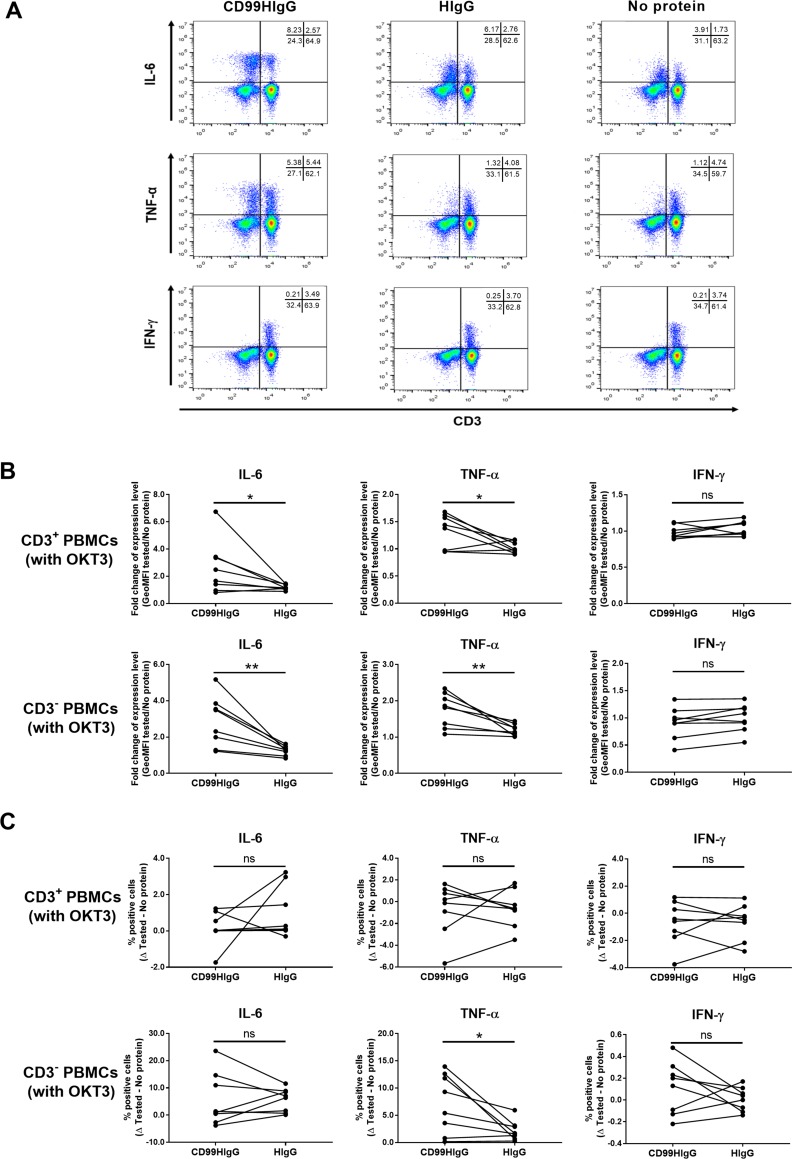
Flow cytometric analysis of intracellular cytokines in OKT3 stimulated PBMCs in the presence of CD99HIgG. PBMCs were stimulated with anti-CD3 mAb (OKT3) in the presence or absence of CD99HIgG or HIgG control. (A) Intracellular IL-6, TNF-α and IFN-γ cytokine profile of CD3^+^ and CD3^-^ cells gated from PBMCs are shown in dot plot. The profile of one of the eight healthy donors is shown. (B) The fold changes of geometric mean fluorescence intensity (GeoMFI) of indicated cytokine positive cells in each tested condition (compared to no added protein condition; no protein) are shown (n = 8). (C) The subtraction of percentage of cytokine expressing cells (% positive cells in tested–no protein) in each tested condition are shown (n = 8). Each dot represents each tested subject and the horizontal lines connect between each subject. Statistical analysis was carried out by unpaired t test. *p ≤ 0.05, **p ≤ 0. 01, ns = not statistically significant.

PBMCs were activated with anti-CD3 mAb OKT3 in the presence or absence of CD99HIgG or purified HIgG control proteins. The intracellular pro-inflammatory cytokines, IL-6, TNF-α and IFN-γ, were determined in CD3^+^ and CD3^-^ mononuclear cell populations. Upon OKT3 activation, in the presence of CD99HIgG but not HIgG control, upregulation of IL-6 and TNF-α expressions (level of intracellular cytokine) in both CD3^+^ and CD3^-^ mononuclear cell populations were observed (Fig 2A and 2B). Meanwhile, IFN-γ expression was not affected by CD99HIgG compared to control (Fig 2A and 2B). In both CD3^+^ and CD3^-^ mononuclear cell populations, the frequency of IL-6 or IFN-γ producing cells (% cytokine producing cells) in the presence of CD99HIgG was not statistically significantly different compared to controls (Fig 2A and 2C). The frequency of TNF-α producing cells was increased only in CD3^-^ mononuclear cells, but not CD3^+^ T cells, in the presence of CD99HIgG compared with control proteins (Fig 2A and 2C). Additionally, there was no statistically significant difference in either level or frequency of IL-4 and IL-10 production by CD3^+^ T cells in comparison between the presence of CD99HIgG and control (S1 Fig).

From the obtained results, we hypothesized that CD99 ligands might be expressed on cell surface. The interaction between CD99 and its ligand plays an important role in the induction of, at least, IL-6 and TNF-α production, particularly in CD3^-^ mononuclear cell population.

**Fig 3:**
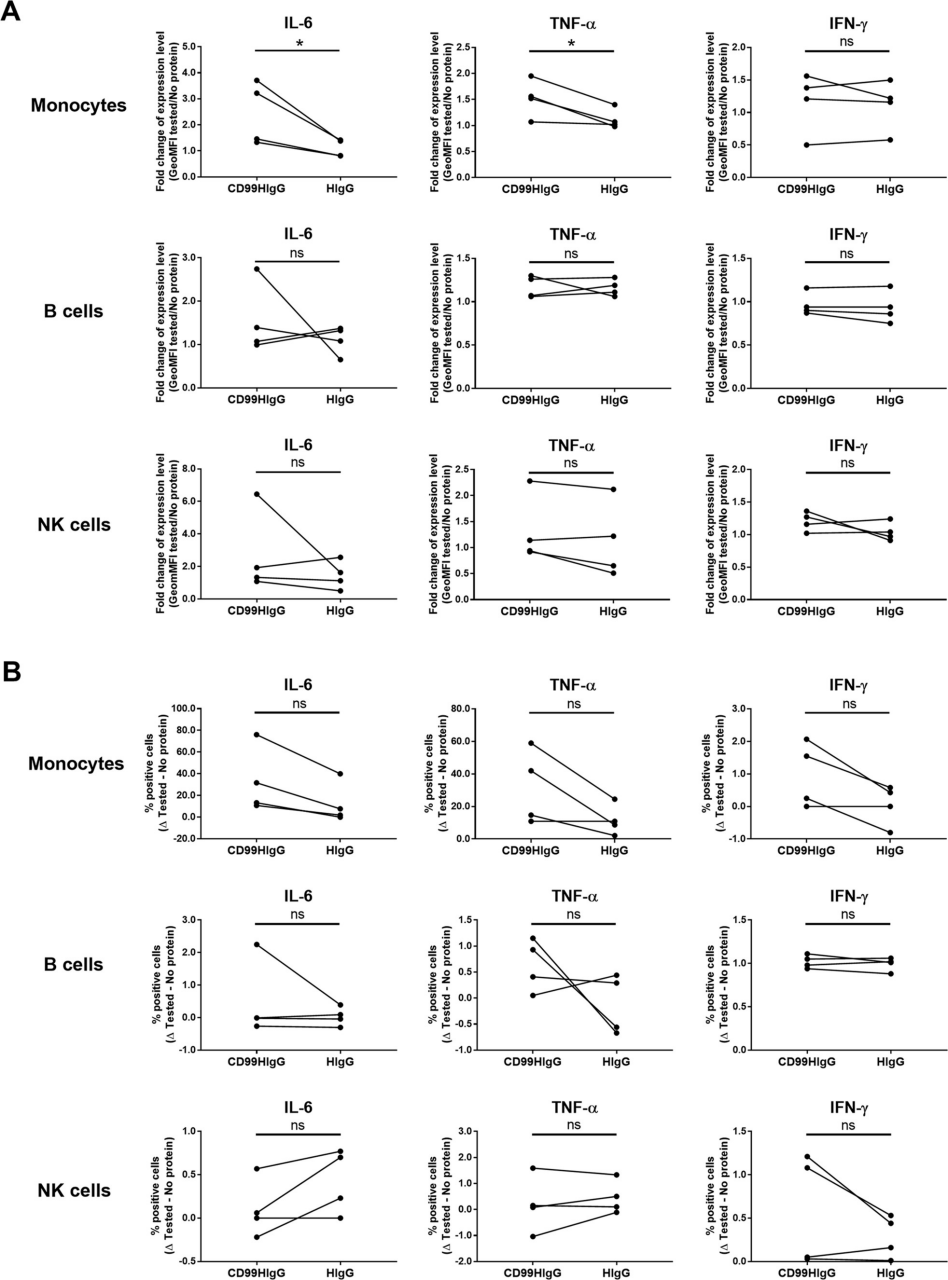
Flow cytometric analysis of intracellular cytokines produced by monocytes, B cells and NK cells upon OKT3 stimulated PBMCs in the presence of CD99HIgG. PBMCs were stimulated with anti-CD3 mAb (OKT3) in the presence or absence of CD99HIgG or HIgG control. Cells were gated for the determination of intracellular IL-6, TNF-α and IFN-γ in monocytes, B cells and NK cells. The gating strategies for monocytes, B cells and NK cells were shown in S2 Fig. (A) The graphs indicate fold change of geometric mean fluorescence intensity (GeoMFI) (compared to no added protein condition) of cytokine positive cells in the indicated conditions (n = 4). (B) The graphs indicate the subtraction of percentage of cytokine positive cells (% positive cells in tested–no protein) in the indicated conditions (n = 4). Each dot represents each tested subject and the horizontal lines connect between each subject. Statistical analysis was performed by unpaired t test. *p ≤ 0.05; ns = not statistically significant.

Consequently, we further clarified the CD3^-^ cell subpopulations that increased in IL-6 and TNF-α production in response to CD99. Upon OKT3 activation, in the presence of CD99HIgG, the expression level of IL-6 and TNF-α in monocytes but not in B and NK cells was significantly increased compared with HIgG control (Fig 3A). However, in all CD3^-^ cell subpopulations, the frequency of IL-6 and TNF-α producing cells (% cytokine producing cells) was not statistically significantly different (Fig 3B). The IFN-γ expression by all CD3^-^ cell subpopulations tested was not affected by CD99HIgG compared to control (Fig 3A and 3B). These results suggested that the interaction between CD99 and its ligand expressed on monocytes, but not B and NK cells, plays an important role in the induction of, at least, IL-6 and TNF-α production.

### Interaction of CD99 and its ligand on monocytes and NK cells upregulates IL-6 and TNF-α production in unstimulated condition

**Fig 4:**
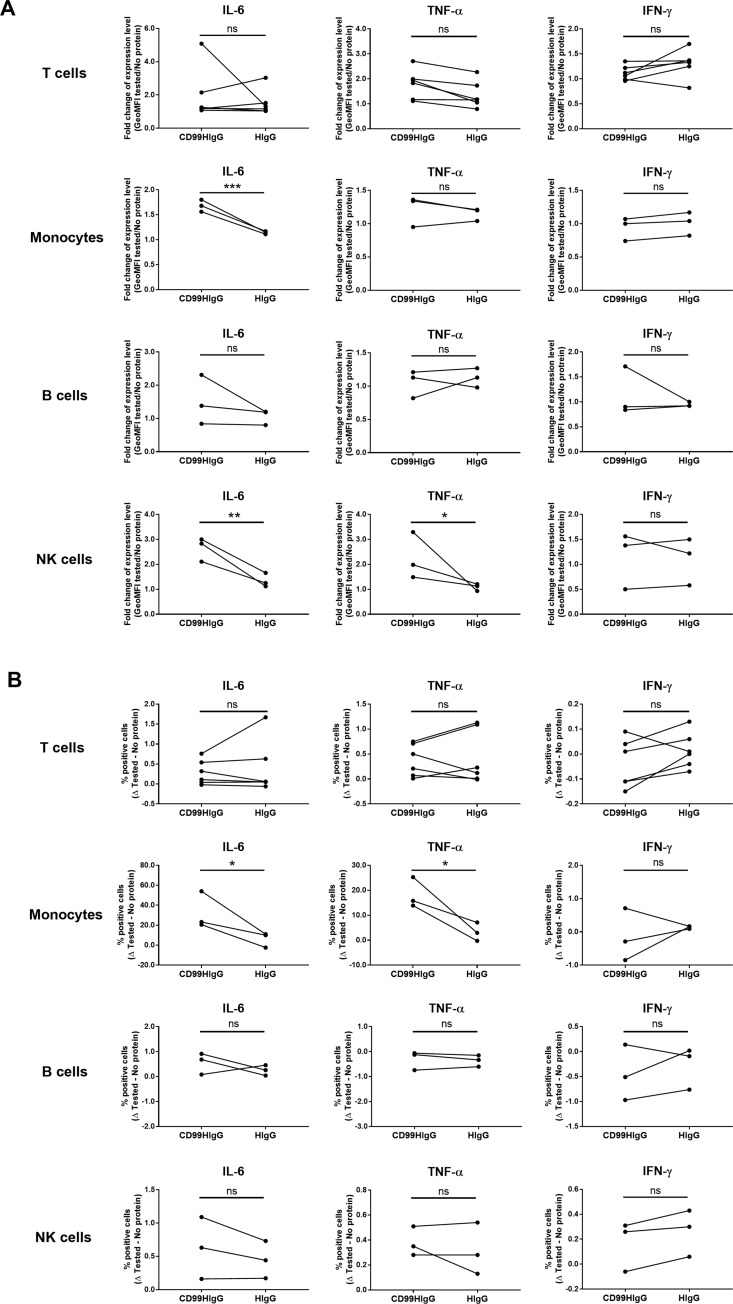
Flow cytometric analysis of intracellular cytokines in unstimulated PBMCs in the presence of CD99HIgG. PBMCs were cultured in the presence or absence of CD99HIgG or HIgG control. T cells, monocytes, B cells and NK cells were gated for the determination of intracellular IL-6, TNF-α and IFN-γ production. The gating strategies were showed in S2 Fig. (A) The graphs indicate fold change of geometric mean fluorescence intensity (GeoMFI) of cytokine positive cells in the indicated conditions (n = 6 for T cells, n = 3 for monocytes, B cells and NK cells). (B) The graphs indicate the subtraction of percentage of cytokine positive cells in the indicated conditions (n = 6 for T cells, n = 3 for monocytes, B cells and NK cells). Each dot represents each tested subject and the horizontal lines connect between each subject. Statistical analysis was performed by unpaired t test. *p ≤ 0.05, **p ≤ 0. 01, ***p ≤ 0. 001, ns = not statistically significant.

From the results that the interaction of CD99 and its ligand regulated IL-6 and TNF-α production upon T cell activation, we further investigated whether this effect occurred in unstimulated condition. PBMCs were treated with or without CD99HIgG or HIgG control proteins without OKT3 stimulation. In CD3^+^ T cells, the level (Fig 4A) and the frequency (Fig 4B) of intracellular IL-6, TNF-α, and IFN-γ expression were not significantly altered in the presence of CD99HIgG compared to HIgG control. The results suggested that the upregulation of IL-6 and TNF-α production by the recombinant CD99 observed in T cells (Fig 2) required the signal from T cell activation.

In the monocytes, in comparison between CD99HIgG and HIgG control, the level of IL-6 expression but not for TNF-α and IFN-γ was statistically different (Fig 4A). An increase in IL-6 as well as TNF-α producing cells, however, could be observed (Fig 4B). In NK cells, the production of IL-6 and TNF-α was increased in the expression levels but not for the frequency of positive cells (Fig 4A and 4B). In contrast, the difference in all tested cytokines, both in level and frequency, was not observed in B cells (Fig 4A and 4B). It is worth mentioning that in all analyzed cells, the IFN-γ expression was not affected by CD99HIgG compared to control (Fig 4A and 4B).

Taken together, the results suggested that the direct interaction of CD99 and its ligand regulated the IL-6 and TNF-α production on monocytes and NK cells.

### Interaction of CD99 and its ligand has no effect on T cell proliferation and expression of activation-associated markers

**Fig 5:**
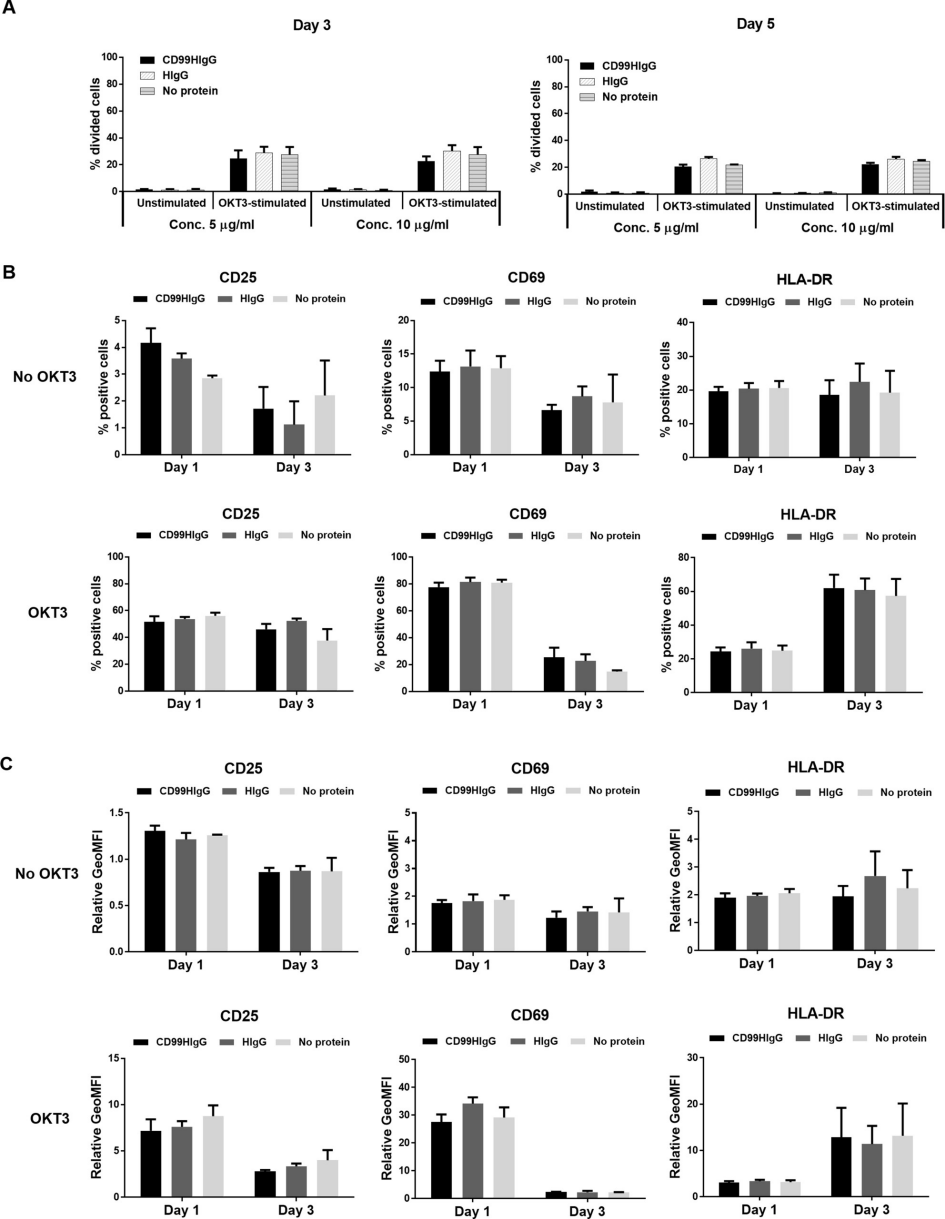
Effect of CD99HIgG on T cell proliferation and activation-associated markers expression. (A) CFSE-labeled PBMCs were stimulated with or without sub-optimal dose of anti-CD3 mAb (OKT3) in the presence or absence of CD99HIgG or HIgG control (5 and 10 μg/ml). The percentage of divided cells (mean±SE; n = 3) at day 3 and day 5 is shown in bar graphs. Statistical analysis was tested by one-way ANOVA with multiple comparisons of Sidak. The expressions of CD25 or CD69 or HLA-DR in PBMCs detected at day 1 and day 3 are shown in B and C. The bar graphs of percentage of positive cells (B) and relative geometric mean fluorescence intensity (GeoMFI of tested Abs/GeoMFI of isotype-matched control Abs) (C) of PBMCs are exhibited. This data represents mean±SE of three healthy donors. Statistical analysis was achieved by one-way ANOVA with multiple comparisons of Sidak.

We further determined whether the interaction of CD99 and its ligand has an effect on T cell proliferation. CFSE-labeled PBMCs were stimulated with sub-optimal dose of anti-CD3 mAb (OKT3) or kept unstimulated in the presence of CD99HIgG or HIgG control. Cell proliferation was investigated at day 3 and day 5. It was found that no significant difference was observed between in the presence of 5 and 10 μg/ml of CD99HIgG or HIgG proteins, in both OKT3 stimulation and non-stimulation conditions (Fig 5A).

We also determined the effect of CD99 and its ligand interaction on the expression of activation-associated markers, CD25 and CD69 as well as MHC class II. As shown in Fig 5B and 5C, the percentage of positive cells and the expression level of the tested activation-associated markers was not significantly different in the presence of CD99HIgG compared to HIgG control proteins.

### CD99 ligands are expressed on surface of monocytes, NK cells and dendritic cells, but not on B and T cells

**Fig 6:**
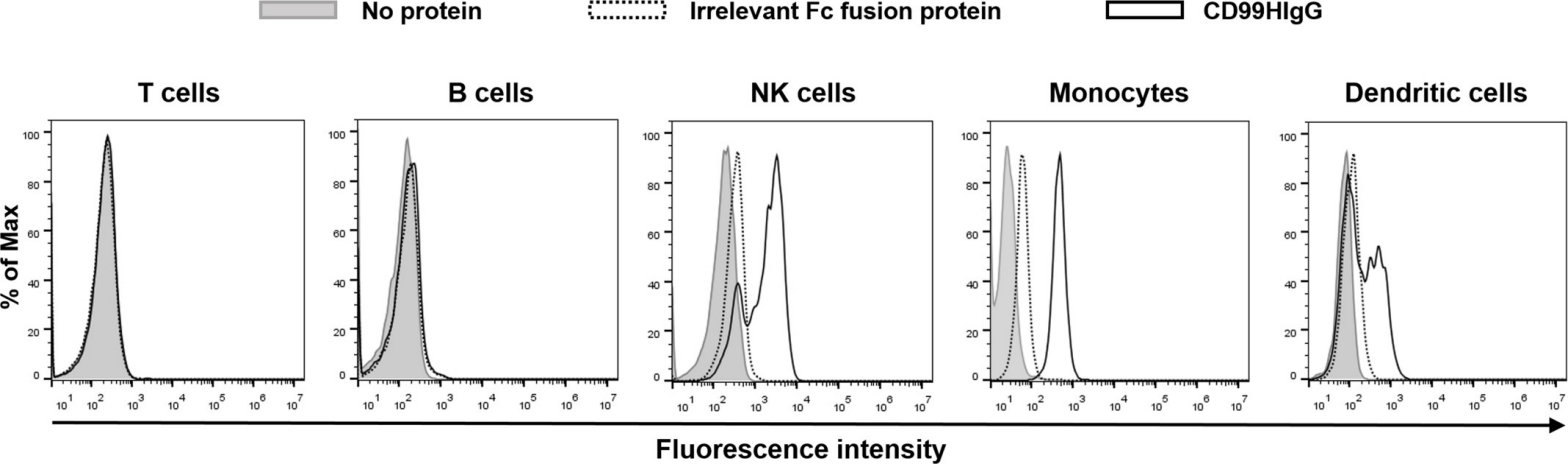
Determination of the expression of CD99 ligands. Fc receptor blocked PBMCs were stained with biotin-labeled CD99HIgG or CD147ExHIgG control then crosslinked with DTSSP. PBMCs were stained with antibodies specifically reacted to cell surface marker for analysis of T cells, B cells, monocytes, NK cells and dendritic cells together with streptavidin-PE. The gating strategies were showed in S3 Fig. Streptavidin-PE positive cells were determined in each cell subpopulation. The overlayered histograms in the indicated cell populations are shown. The figure is a representative result from one of the three individuals. No protein staining (gray peak), CD147ExHIgG, an irrelevant Fc fusion protein, (dot line) and CD99HIgG (solid line).

Regarding our results, we speculated that CD99 ligands exist and is expressed on the surface of any peripheral blood mononuclear cell population. To confirm this speculation, Fc receptor blocked PBMCs were stained with CD99HIgG or irrelevant Fc fusion protein (CD147ExHIgG). DTSSP were used to covalent link between CD99HIgG and its ligands. As shown in Fig 6, CD99HIgG specifically bound to monocytes, NK cells and dendritic cells, but not on B and T lymphocytes.

## Discussion

CD99 is a transmembrane glycoprotein expressed on various cell types [11, 12, 37]. Several functions of CD99 molecules have been reported, including the involvement in T cell activation [18–20, 38]. Co-ligation of CD3 and CD99 with agonistic antibodies induced translocation of T cell receptor (TCR), ζ and ε chains into lipid rafts and amplification of TCR signaling by which this mechanism differs from the activation of CD28 co-stimulatory molecule [29]. Stimulation with anti-CD99 and anti-CD3 mAbs without anti-CD28 mAb could induce T cell proliferation, CD25 upregulation and Th1 cytokine production [22, 39]. Controversially, the inhibition of T cell activation by anti-CD99 mAb has also been reported [28]. The functions of CD99 reported, however, were determined by employing specific mAb to mimic the binding of CD99 to its ligands expressed on cell surface. This made us imagine that CD99 ligands remain and play a role in immunoregulation.

In the present study, we demonstrated the presence of CD99 ligands on leukocyte surface and its interaction with CD99 induced IL-6 and TNF-α production. Soluble recombinant CD99-IgG Fc fusion proteins, CD99HIgG, were firstly produced and used as a tool in this study. For recombinant CD99 production, human cell line was used as the producing cells to gain post-translational modification of the recombinant CD99 proteins similar to their native form [40, 41]. Human IgG Fc tag was fused with extracellular part of CD99 for facilitation of the purification process. The generated recombinant CD99 molecules were designed to contain only extracellular part of CD99 without the transmembrane part. This, therefore, leads to the secretion of CD99HIgG for large-scale production and purification. Importantly, CD99HIgG was designed to contain a hinge region for flexibility in binding and ligand clustering. The CD99HIgG was then used to investigate its effect upon binding to CD99 ligands.

Upon T cell activation, CD99HIgG induced the upregulation of IL-6 and TNF-α expression in CD3^+^ T cells. Without T cell activation, in contrast, no cytokines were upregulated. These results suggested that the effect of CD99-CD99 ligand interaction on upregulation of the cytokine production in T cells requires the signal(s) from T cell activation. However, the upregulation of IL-6 and TNF-α expression was observed in non-T cell population either with or without T cell activation. Although the precise mechanism is still unknown, the results suggested that CD99 ligands are in existence and the interaction with CD99 is involved in the regulation of pro-inflammatory cytokine production. CD99 ligands expressed on immune cells probably have a crucial function in immune response and inflammation [42–45].

Upon T cell activation, several activation-associated molecules, including CD25, CD69 and HLA-DR are upregulated at specific time periods, ultimately resulting in T cell proliferation [46, 47]. After T cell triggering, CD25, an IL-2 receptor α-chain (IL-2Rα), is expressed within 24 h and form a complex with two polypeptide chains, IL-2Rβ and IL-2Rγc. The trimeric IL-2Rαβγc is a high affinity receptor for IL-2. Interaction between IL-2 and IL-2R plays a critical role in T cell proliferation [48]. The CD69 is a phosphorylated disulfide-linked 28- to 32-kDa homodimer. CD69 has a rapid onset (within 2 h) of expression after T cell activation [49]. The stimulation of CD69 induces IL-2 gene expression. The CD25 upregulation during T cell activation leading to CD69-induced T cell proliferation is IL-2/CD25 dependent [50]. MHC class II (HLA-DR) is a late marker that is expressed at 48–72 h after T cell activation. Its expression is correlated with the ability of cells to grow in long term culture [51]. These three T cells activating markers were, therefore, selected in this study in order to determine the effect of CD99 and its ligand interaction. In our study, however, CD99 and its ligand interaction did not alter the expression of all tested molecules. Consequently, cell proliferation was not affected by CD99 and its ligand interaction. Taken together, our results demonstrated that CD99 and its ligand interaction selectively induce cytokine production during T cell activation. The induction of pro-inflammatory cytokine synthesis following the interaction between T cells and monocytes, mediated by specific surface molecules such as CD40L/CD40 and ICAM/LFA-1 interaction, has been reported [52–55]. We hypothesized that the binding of CD99 and its ligands might be another pair molecule that regulated pro-inflammatory cytokine production.

We further investigated the CD99 ligand expression on peripheral blood mononuclear cells. The CD99 ligands were demonstrated to express on monocytes, NK cells and dendritic cells. No CD99 ligand was observed on the surface of B and T cells. The CD99 molecules were reported to be extensively expressed on activated/memory T cells [56, 57] and are required to bind to their ligands that are expressed on antigen presenting cells (APCs). Although, the mechanisms of CD99 and its ligand interaction in regulation pro-inflammatory cytokines production have not been defined in this study, we hypothesized that binding of CD99 to its ligands on APCs and/or to its ligands on T cells which were expressed upon T cell activation may lead to the alteration of cytokine production. Hence, CD99 ligands are further needed to be identified so that the insight mechanisms will be clarified.

## Conclusions

In conclusion, we report here the presence of CD99 ligands on leukocyte surface. Interaction between CD99 and its ligand mediates the upregulation of pro-inflammatory cytokine expression. The interaction between CD99 and its ligands is probably involved in the inflammatory condition occurring in the body. Blockage of CD99 and its ligand may abolish the production of inflammatory cytokines and may be a new strategy for the treatment of inflammatory diseases.

## Supporting information

S1 Fig Analysis of the effect of CD99HIgG on IL-4 and IL-10 productions. PBMCs were stimulated with anti-CD3 mAb (OKT3) in the presence or absence of CD99HIgG or HIgG control. Intracellular IL-4 and IL-10 expression of CD3^+^ T cells gated from PBMCs were determined. (A) The fold changes of geometric mean fluorescence intensity (GeoMFI) of indicated cytokine positive cells in each tested condition are shown (n = 3). (B) The subtraction of percentage of cytokine expressing cells (% positive cells in tested–no protein) in each tested condition are shown (n = 3). Each dot represents each tested subject and the horizontal lines connect between each subject. Statistical analysis was carried out by unpaired t test. ns = not statistically significant.
(TIF)

S2 Fig Flow cytometric gating strategy for analysis of intracellular cytokine expression. Size (forward scatter; FSC) and granularity (side scatter; SSC) of peripheral blood mononuclear cells (PBMCs) were plotted and used for cell gating as indicated. (A) The gated cells were plotted against side scatter (SSC) and CD14. Monocytes were discriminated from lymphocytes based on CD14 expression and then CD14^+^ monocytes were further plotted against cytokine expression and CD14. (B) The gated cells were plotted against CD3 and CD19 and then CD3^-^CD19^+^ B cells were further plotted against cytokine expression and CD19. (C) The gated cells were plotted against CD3 and CD56 and then CD3^-^CD56^+^ NK cells were further plotted against cytokine expression and CD56. The cytokine expression in term of level of expression and frequency in each population were investigated.
(TIF)

S3 Fig Flow cytometric gating strategy for analysis of CD99 ligand expression. Size (forward scatter; FSC) and granularity (side scatter; SSC) of peripheral blood mononuclear cells (PBMCs) were plotted and used for cell gating as indicated. (A) The gated cells were plotted against CD3 and CD56. The CD3^+^CD56^-^ T cell and CD3^-^CD56^+^ NK cells were further gated. (B) The gated cells were plotted against CD14 and CD19. CD14^+^ monocytes and CD19^+^ B cells were further gated. (C) Dendritic cells were identified by CD3^-^CD14^-^CD16^-^CD19^-^CD56^-^ and HLA-DR^+^ cells. The gated cells were plotted against CD56 and CD3, CD14, CD16, CD19 for lineage negative cell gating. The lineage negative gated cells were plotted against SSC and HLA-DR and dendritic cells were further gated. In each gated population (i.e. NK cells, T cells, Monocytes, B cells and dendritic cells), the percentage of phycoerythrin (PE) positive cells were investigated.
(TIF)
