# Supplementary figures and images for: Interaction of CD99 and its ligand upregulates IL-6 and TNF-α upon T cell activation
Source: PLoS One. 2019 May 23;14(5):e0217393. doi: 10.1371/journal.pone.0217393 (PMC6532917; doi:10.1371/journal.pone.0217393)

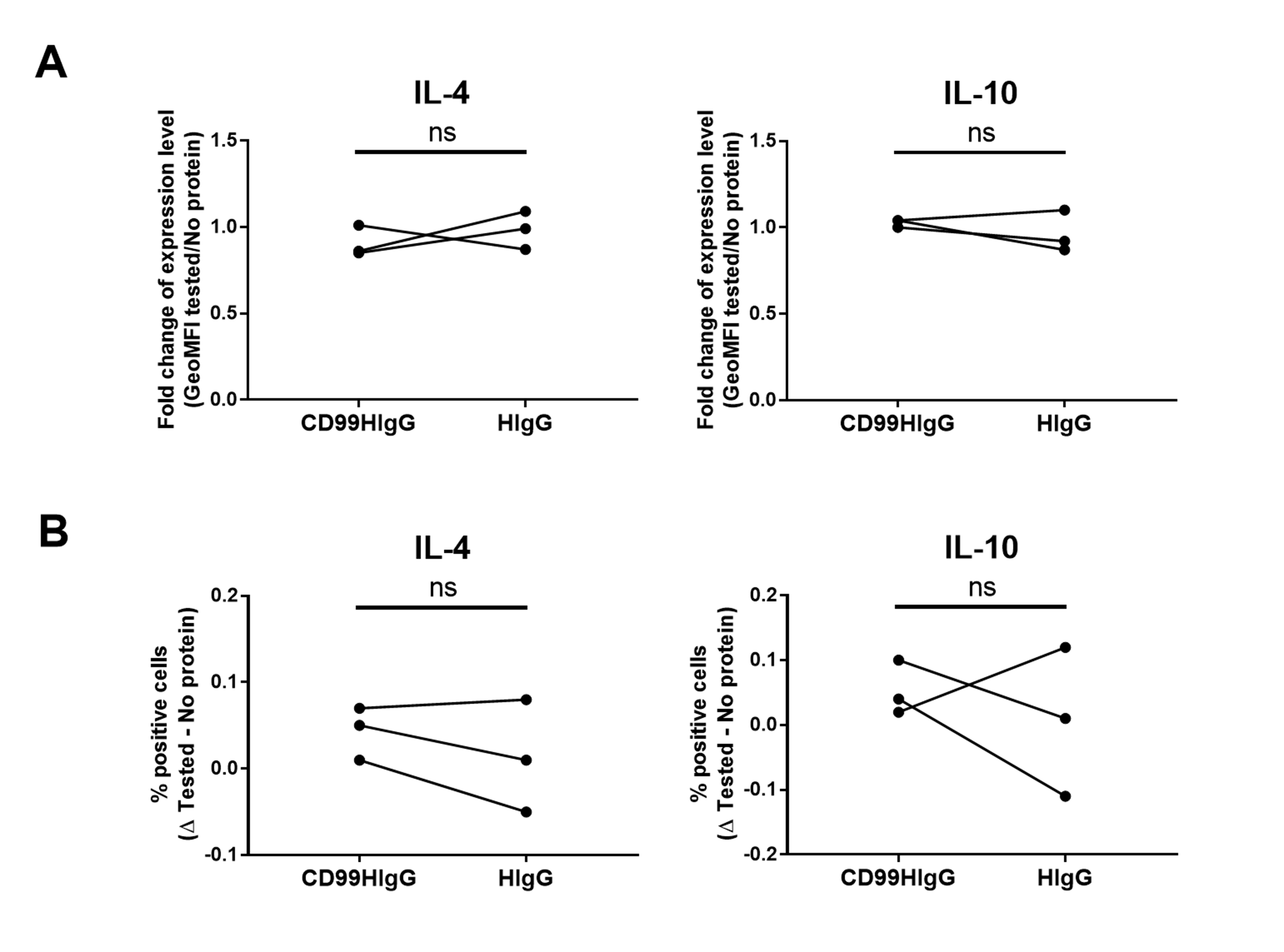

Supplement: S1 Fig — PBMCs were stimulated with anti-CD3 mAb (OKT3) in the presence or absence of CD99HIgG or HIgG control. Intracellular IL-4 and IL-10 expression of CD3+ T cells gated from PBMCs were determined. (A) The fold changes of geometric mean fluorescence intensity (GeoMFI) of indicated cytokine positive cells in each tested condition are shown (n = 3). (B) The subtraction of percentage of cytokine expressing cells (% positive cells in tested–no protein) in each tested condition are shown (n = 3). Each dot represents each tested subject and the horizontal lines connect between each subject. Statistical analysis was carried out by unpaired t test. ns = not statistically significant. (TIF) [file pone.0217393.s001.tif]

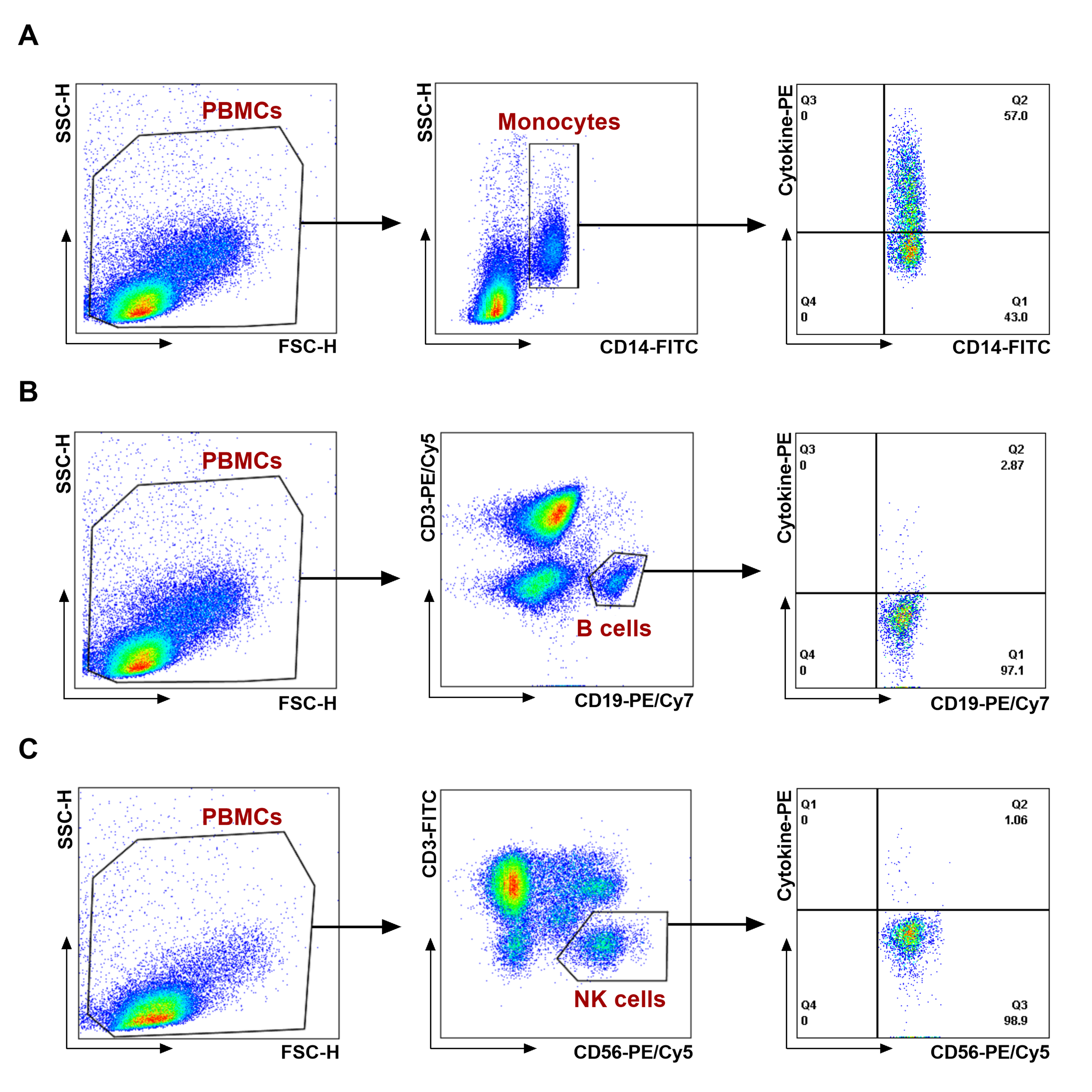

Supplement: S2 Fig — Size (forward scatter; FSC) and granularity (side scatter; SSC) of peripheral blood mononuclear cells (PBMCs) were plotted and used for cell gating as indicated. (A) The gated cells were plotted against side scatter (SSC) and CD14. Monocytes were discriminated from lymphocytes based on CD14 expression and then CD14+ monocytes were further plotted against cytokine expression and CD14. (B) The gated cells were plotted against CD3 and CD19 and then CD3-CD19+ B cells were further plotted against cytokine expression and CD19. (C) The gated cells were plotted against CD3 and CD56 and then CD3-CD56+ NK cells were further plotted against cytokine expression and CD56. The cytokine expression in term of level of expression and frequency in each population were investigated. (TIF) [file pone.0217393.s002.tif]

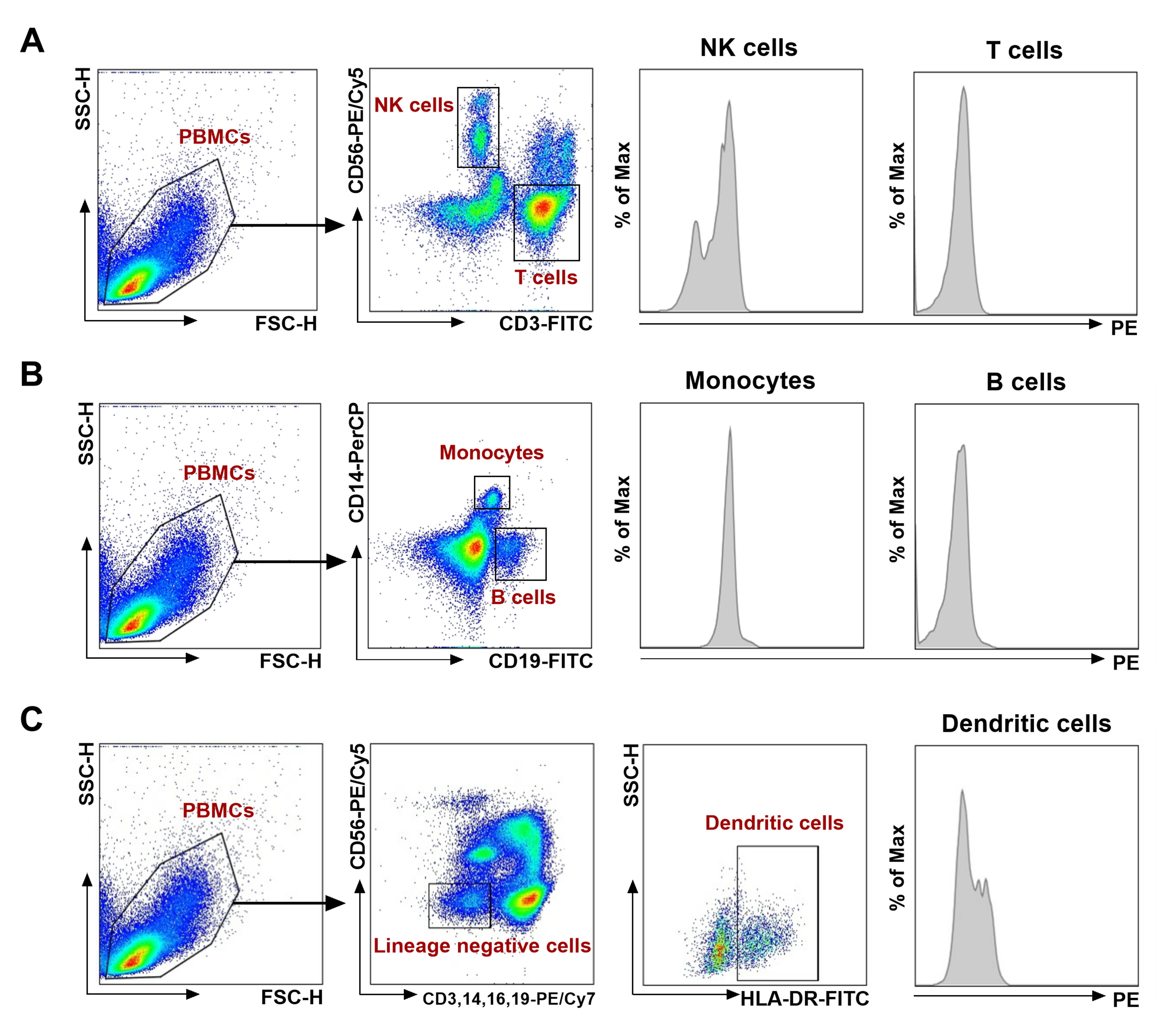

Supplement: S3 Fig — Size (forward scatter; FSC) and granularity (side scatter; SSC) of peripheral blood mononuclear cells (PBMCs) were plotted and used for cell gating as indicated. (A) The gated cells were plotted against CD3 and CD56. The CD3+CD56- T cell and CD3-CD56+ NK cells were further gated. (B) The gated cells were plotted against CD14 and CD19. CD14+ monocytes and CD19+ B cells were further gated. (C) Dendritic cells were identified by CD3-CD14-CD16-CD19-CD56- and HLA-DR+ cells. The gated cells were plotted against CD56 and CD3, CD14, CD16, CD19 for lineage negative cell gating. The lineage negative gated cells were plotted against SSC and HLA-DR and dendritic cells were further gated. In each gated population (i.e. NK cells, T cells, Monocytes, B cells and dendritic cells), the percentage of phycoerythrin (PE) positive cells were investigated. (TIF) [file pone.0217393.s003.tif]
